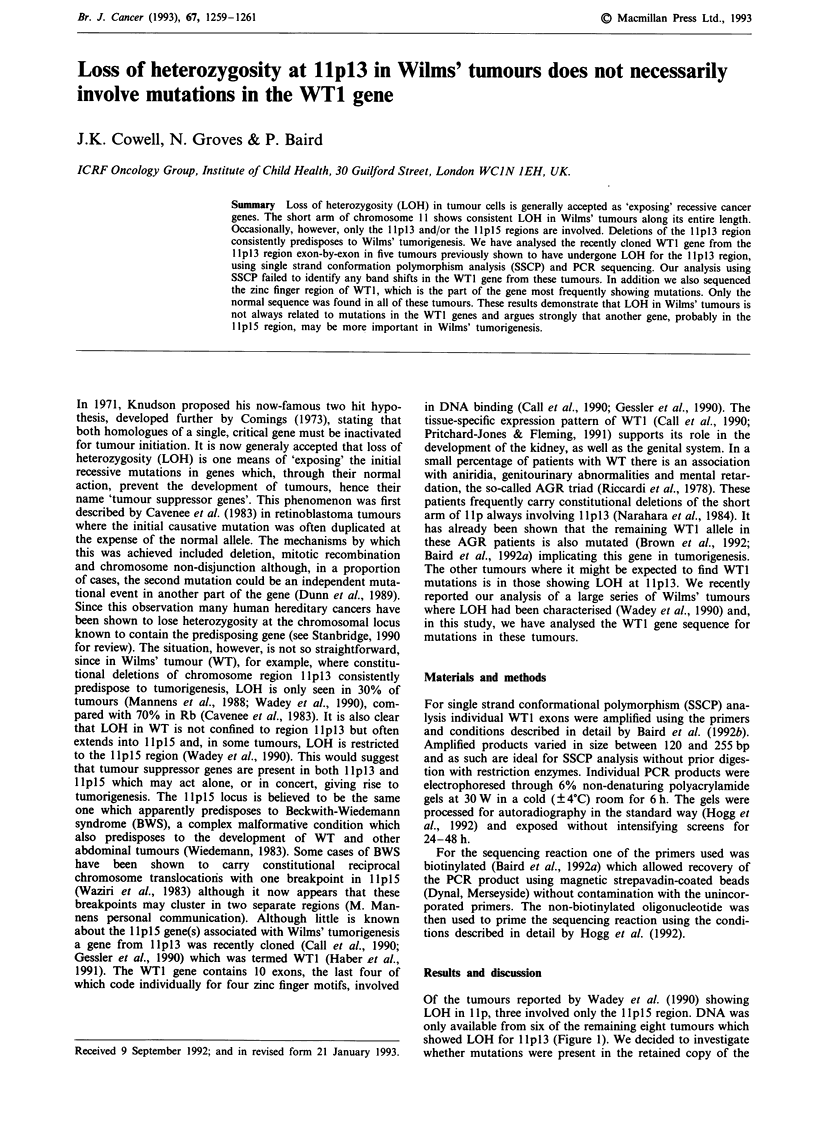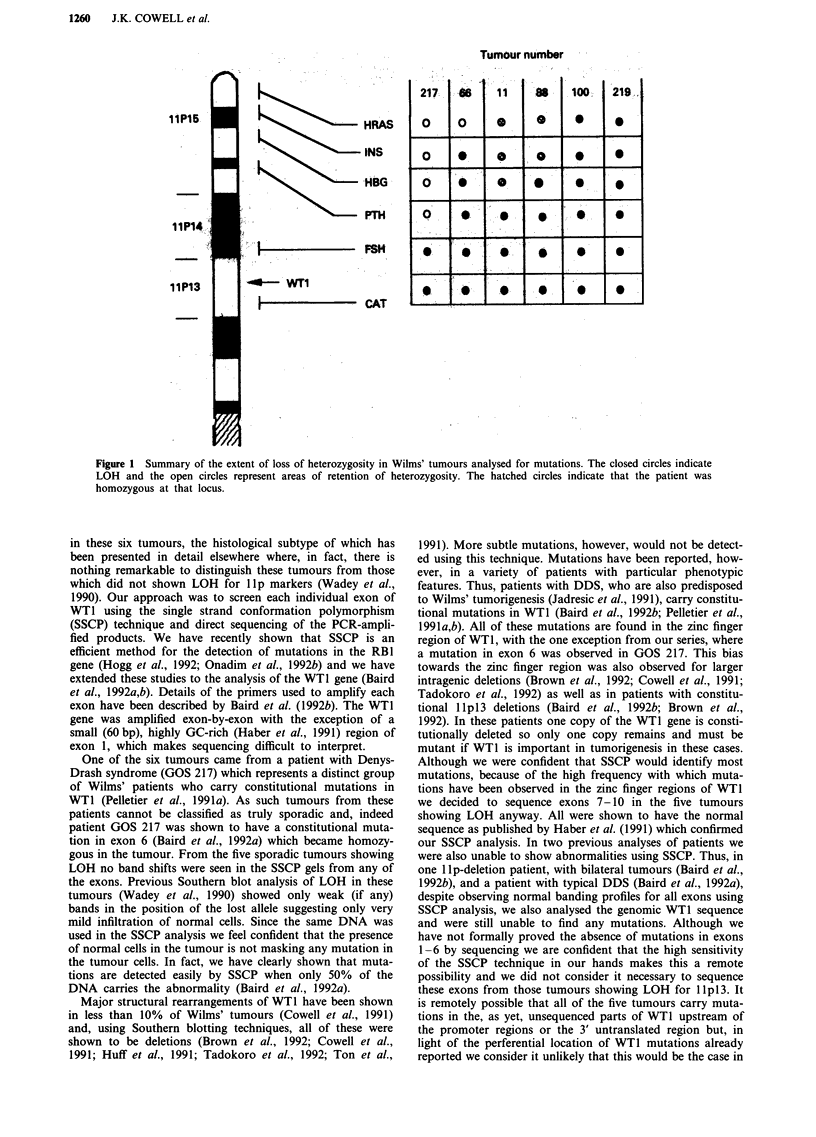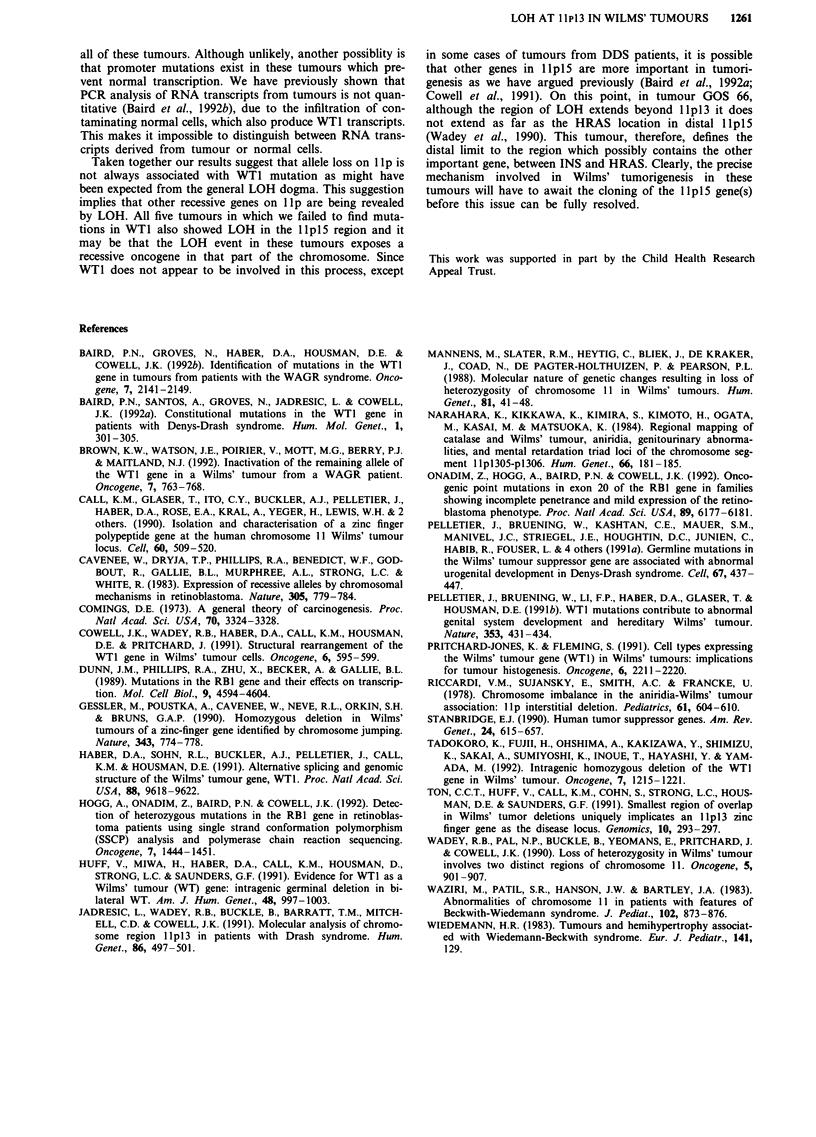# Loss of heterozygosity at 11p13 in Wilms' tumours does not necessarily involve mutations in the WT1 gene.

**DOI:** 10.1038/bjc.1993.235

**Published:** 1993-06

**Authors:** J. K. Cowell, N. Groves, P. Baird

**Affiliations:** ICRF Oncology Group, Institute of Child Health, London, UK.

## Abstract

Loss of heterozygosity (LOH) in tumour cells is generally accepted as 'exposing' recessive cancer genes. The short arm of chromosome 11 shows consistent LOH in Wilms' tumours along its entire length. Occasionally, however, only the 11p13 and/or the 11p15 regions are involved. Deletions of the 11p13 region consistently predisposes to Wilms' tumorigenesis. We have analysed the recently cloned WT1 gene from the 11p13 region exon-by-exon in five tumours previously shown to have undergone LOH for the 11p13 region, using single strand conformation polymorphism analysis (SSCP) and PCR sequencing. Our analysis using SSCP failed to identify any band shifts in the WT1 gene from these tumours. In addition we also sequenced the zinc finger region of WT1, which is the part of the gene most frequently showing mutations. Only the normal sequence was found in all of these tumours. These results demonstrate that LOH in Wilms' tumours is not always related to mutations in the WT1 genes and argues strongly that another gene, probably in the 11p15 region, may be more important in Wilms' tumorigenesis.


Br. J. Cancer (1993), 67, 1259–1261 © Macmillan Press Ltd., 1993

### Loss of heterozygosity at 11p13 in Wilms' tumours does not necessarily involve mutations in the WT1 gene

J.K. Cowell, N. Groves & P. Baird

*ICRF Oncology Group, Institute of Child Health, 30 Guilford Street, London WC1N 1EH, UK.*

**Summary** Loss of heterozygosity (LOH) in tumour cells is generally accepted as 'exposing' recessive cancer genes. The short arm of chromosome 11 shows consistent LOH in Wilms' tumours along its entire length. Occasionally, however, only the 11p13 and/or the 11p15 regions are involved. Deletions of the 11p13 region consistently predisposes to Wilms' tumorigenesis. We have analysed the recently cloned WT1 gene from the 11p13 region exon-by-exon in five tumours previously shown to have undergone LOH for the 11p13 region, using single strand conformation polymorphism analysis (SSCP) and PCR sequencing. Our analysis using SSCP failed to identify any band shifts in the WT1 gene from these tumours. In addition we also sequenced the zinc finger region of WT1, which is the part of the gene most frequently showing mutations. Only the normal sequence was found in all of these tumours. These results demonstrate that LOH in Wilms' tumours is not always related to mutations in the WT1 genes and argues strongly that another gene, probably in the 11p15 region, may be more important in Wilms' tumorigenesis.

In 1971, Knudson proposed his now-famous two hit hypothesis, developed further by Comings (1973), stating that both homologues of a single, critical gene must be inactivated for tumour initiation. It is now generaly accepted that loss of heterozygosity (LOH) is one means of 'exposing' the initial recessive mutations in genes which, through their normal action, prevent the development of tumours, hence their name 'tumour suppressor genes'. This phenomenon was first described by Cavenee *et al.* (1983) in retinoblastoma tumours where the initial causative mutation was often duplicated at the expense of the normal allele. The mechanisms by which this was achieved included deletion, mitotic recombination and chromosome non-disjunction although, in a proportion of cases, the second mutation could be an independent mutational event in another part of the gene (Dunn *et al.*, 1989). Since this observation many human hereditary cancers have been shown to lose heterozygosity at the chromosomal locus known to contain the predisposing gene (see Stanbridge, 1990 for review). The situation, however, is not so straightforward, since in Wilms' tumour (WT), for example, where constitutional deletions of chromosome region 11p13 consistently predispose to tumorigenesis, LOH is only seen in 30% of tumours (Mannens *et al.*, 1988; Wadey *et al.*, 1990), compared with 70% in Rb (Cavenee *et al.*, 1983). It is also clear that LOH in WT is not confined to region 11p13 but often extends into 11p15 and, in some tumours, LOH is restricted to the 11p15 region (Wadey *et al.*, 1990). This would suggest that tumour suppressor genes are present in both 11p13 and 11p15 which may act alone, or in concert, giving rise to tumorigenesis. The 11p15 locus is believed to be the same one which apparently predisposes to Beckwith-Wiedemann syndrome (BWS), a complex malformative condition which also predisposes to the development of WT and other abdominal tumours (Wiedemann, 1983). Some cases of BWS have been shown to carry constitutional reciprocal chromosome translocations with one breakpoint in 11p15 (Waziri *et al.*, 1983) although it now appears that these breakpoints may cluster in two separate regions (M. Mannens personal communication). Although little is known about the 11p15 gene(s) associated with Wilms' tumorigenesis a gene from 11p13 was recently cloned (Call *et al.*, 1990; Gessler *et al.*, 1990) which was termed WT1 (Haber *et al.*, 1991). The WT1 gene contains 10 exons, the last four of which code individually for four zinc finger motifs, involved in DNA binding (Call *et al.*, 1990; Gessler *et al.*, 1990). The tissue-specific expression pattern of WT1 (Call *et al.*, 1990; Pritchard-Jones & Fleming, 1991) supports its role in the development of the kidney, as well as the genital system. In a small percentage of patients with WT there is an association with aniridia, genitourinary abnormalities and mental retardation, the so-called AGR triad (Riccardi *et al.*, 1978). These patients frequently carry constitutional deletions of the short arm of 11p always involving 11p13 (Narahara *et al.*, 1984). It has already been shown that the remaining WT1 allele in these AGR patients is also mutated (Brown *et al.*, 1992; Baird *et al.*, 1992*a*) implicating this gene in tumorigenesis. The other tumours where it might be expected to find WT1 mutations is in those showing LOH at 11p13. We recently reported our analysis of a large series of Wilms' tumours where LOH had been characterised (Wadey *et al.*, 1990) and, in this study, we have analysed the WT1 gene sequence for mutations in these tumours.

Received 9 September 1992; and in revised form 21 January 1993.

### Materials and methods

For single strand conformational polymorphism (SSCP) analysis individual WT1 exons were amplified using the primers and conditions described in detail by Baird *et al.* (1992*b*). Amplified products varied in size between 120 and 255 bp and as such are ideal for SSCP analysis without prior digestion with restriction enzymes. Individual PCR products were electrophoresed through 6% non-denaturing polyacrylamide gels at 30 W in a cold (±4°C) room for 6 h. The gels were processed for autoradiography in the standard way (Hogg *et al.*, 1992) and exposed without intensifying screens for 24–48 h.

For the sequencing reaction one of the primers used was biotinylated (Baird *et al.*, 1992*a*) which allowed recovery of the PCR product using magnetic strepavadin-coated beads (Dynal, Merseyside) without contamination with the unincorporated primers. The non-biotinylated oligonucleotide was then used to prime the sequencing reaction using the conditions described in detail by Hogg *et al.* (1992).

### Results and discussion

Of the tumours reported by Wadey *et al.* (1990) showing LOH in 11p, three involved only the 11p15 region. DNA was only available from six of the remaining eight tumours which showed LOH for 11p13 (Figure 1). We decided to investigate whether mutations were present in the retained copy of the

**Figure 1** Summary of the extent of loss of heterozygosity in Wilms' tumours analysed for mutations. The closed circles indicate LOH and the open circles represent areas of retention of heterozygosity. The hatched circles indicate that the patient was homozygous at that locus.

in these six tumours, the histological subtype of which has been presented in detail elsewhere where, in fact, there is nothing remarkable to distinguish these tumours from those which did not shown LOH for 11p markers (Wadey *et al.*, 1990). Our approach was to screen each individual exon of WT1 using the single strand conformation polymorphism (SSCP) technique and direct sequencing of the PCR-amplified products. We have recently shown that SSCP is an efficient method for the detection of mutations in the RB1 gene (Hogg *et al.*, 1992; Onadim *et al.*, 1992*b*) and we have extended these studies to the analysis of the WT1 gene (Baird *et al.*, 1992*a*,*b*). Details of the primers used to amplify each exon have been described by Baird *et al.* (1992*b*). The WT1 gene was amplified exon-by-exon with the exception of a small (60 bp), highly GC-rich (Haber *et al.*, 1991) region of exon 1, which makes sequencing difficult to interpret.

One of the six tumours came from a patient with Denys-Drash syndrome (GOS 217) which represents a distinct group of Wilms' patients who carry constitutional mutations in WT1 (Pelletier *et al.*, 1991*a*). As such tumours from these patients cannot be classified as truly sporadic and, indeed patient GOS 217 was shown to have a constitutional mutation in exon 6 (Baird *et al.*, 1992*a*) which became homozygous in the tumour. From the five sporadic tumours showing LOH no band shifts were seen in the SSCP gels from any of the exons. Previous Southern blot analysis of LOH in these tumours (Wadey *et al.*, 1990) showed only weak (if any) bands in the position of the lost allele suggesting only very mild infiltration of normal cells. Since the same DNA was used in the SSCP analysis we feel confident that the presence of normal cells in the tumour is not masking any mutation in the tumour cells. In fact, we have clearly shown that mutations are detected easily by SSCP when only 50% of the DNA carries the abnormality (Baird *et al.*, 1992*a*).

Major structural rearrangements of WT1 have been shown in less than 10% of Wilms' tumours (Cowell *et al.*, 1991) and, using Southern blotting techniques, all of these were shown to be deletions (Brown *et al.*, 1992; Cowell *et al.*, 1991; Huff *et al.*, 1991; Tadokoro *et al.*, 1992; Ton *et al.*, 1991). More subtle mutations, however, would not be detected using this technique. Mutations have been reported, however, in a variety of patients with particular phenotypic features. Thus, patients with DDS, who are also predisposed to Wilms' tumorigenesis (Jadresic *et al.*, 1991), carry constitutional mutations in WT1 (Baird *et al.*, 1992*b*; Pelletier *et al.*, 1991*a*,*b*). All of these mutations are found in the zinc finger region of WT1, with the one exception from our series, where a mutation in exon 6 was observed in GOS 217. This bias towards the zinc finger region was also observed for larger intragenic deletions (Brown *et al.*, 1992; Cowell *et al.*, 1991; Tadokoro *et al.*, 1992) as well as in patients with constitutional 11p13 deletions (Baird *et al.*, 1992*b*; Brown *et al.*, 1992). In these patients one copy of the WT1 gene is constitutionally deleted so only one copy remains and must be mutant if WT1 is important in tumorigenesis in these cases. Although we were confident that SSCP would identify most mutations, because of the high frequency with which mutations have been observed in the zinc finger regions of WT1 we decided to sequence exons 7–10 in the five tumours showing LOH anyway. All were shown to have the normal sequence as published by Haber *et al.* (1991) which confirmed our SSCP analysis. In two previous analyses of patients we were also unable to show abnormalities using SSCP. Thus, in one 11p-deletion patient, with bilateral tumours (Baird *et al.*, 1992*b*), and a patient with typical DDS (Baird *et al.*, 1992*a*), despite observing normal banding profiles for all exons using SSCP analysis, we also analysed the genomic WT1 sequence and were still unable to find any mutations. Although we have not formally proved the absence of mutations in exons 1–6 by sequencing we are confident that the high sensitivity of the SSCP technique in our hands makes this a remote possibility and we did not consider it necessary to sequence these exons from those tumours showing LOH for 11p13. It is remotely possible that all of the five tumours carry mutations in the, as yet, unsequenced parts of WT1 upstream of the promoter regions or the 3′ untranslated region but, in light of the perferential location of WT1 mutations already reported we consider it unlikely that this would be the case in all of these tumours. Although unlikely, another possiblity is that promoter mutations exist in these tumours which prevent normal transcription. We have previously shown that PCR analysis of RNA transcripts from tumours is not quantitative (Baird *et al.*, 1992*b*), due to the infiltration of contaminating normal cells, which also produce WT1 transcripts. This makes it impossible to distinguish between RNA transcripts derived from tumour or normal cells.

Taken together our results suggest that allele loss on 11p is not always associated with WT1 mutation as might have been expected from the general LOH dogma. This suggestion implies that other recessive genes on 11p are being revealed by LOH. All five tumours in which we failed to find mutations in WT1 also showed LOH in the 11p15 region and it may be that the LOH event in these tumours exposes a recessive oncogene in that part of the chromosome. Since WT1 does not appear to be involved in this process, except in some cases of tumours from DDS patients, it is possible that other genes in 11p15 are more important in tumorigenesis as we have argued previously (Baird *et al.*, 1992*a*; Cowell *et al.*, 1991). On this point, in tumour GOS 66, although the region of LOH extends beyond 11p13 it does not extend as far as the HRAS location in distal 11p15 (Wadey *et al.*, 1990). This tumour, therefore, defines the distal limit to the region which possibly contains the other important gene, between INS and HRAS. Clearly, the precise mechanism involved in Wilms' tumorigenesis in these tumours will have to await the cloning of the 11p15 gene(s) before this issue can be fully resolved.

This work was supported in part by the Child Health Research Appeal Trust.

### References

BAIRD, P.N., GROVES, N., HABER, D.A., HOUSMAN, D.E. & COWELL, J.K. (1992*b*). Identification of mutations in the WT1 gene in tumours from patients with the WAGR syndrome. *Oncogene*, **7**, 2141–2149.

BAIRD, P.N., SANTOS, A., GROVES, N., JADRESIC, L. & COWELL, J.K. (1992*a*). Constitutional mutations in the WT1 gene in patients with Denys-Drash syndrome. *Hum. Mol. Genet.*, **1**, 301–305.

BROWN, K.W., WATSON, J.E., POIRIER, V., MOTT, M.G., BERRY, P.J. & MAITLAND, N.J. (1992). Inactivation of the remaining allele of the WT1 gene in a Wilms' tumour from a WAGR patient. *Oncogene*, **7**, 763–768.

CALL, K.M., GLASER, T., ITO, C.Y., BUCKLER, A.J., PELLETIER, J., HABER, D.A., ROSE, E.A., KRAL, A., YEGER, H., LEWIS, W.H. & 2 others. (1990). Isolation and characterisation of a zinc finger polypeptide gene at the human chromosome 11 Wilms' tumour locus. *Cell*, **60**, 509–520.

CAVENEE, W., DRYJA, T.P., PHILLIPS, R.A., BENEDICT, W.F., GODBOUT, R., GALLIE, B.L., MURPHREE, A.L., STRONG, L.C. & WHITE, R. (1983). Expression of recessive alleles by chromosomal mechanisms in retinoblastoma. *Nature*, **305**, 779–784.

COMINGS, D.E. (1973). A general theory of carcinogenesis. *Proc. Natl Acad. Sci. USA*, **70**, 3324–3328.

COWELL, J.K., WADEY, R.B., HABER, D.A., CALL, K.M., HOUSMAN, D.E. & PRITCHARD, J. (1991). Structural rearrangement of the WT1 gene in Wilms' tumour cells. *Oncogene*, **6**, 595–599.

DUNN, J.M., PHILLIPS, R.A., ZHU, X., BECKER, A. & GALLIE, B.L. (1989). Mutations in the RB1 gene and their effects on transcription. *Mol. Cell Biol.*, **9**, 4594–4604.

GESSLER, M., POUSTKA, A., CAVENEE, W., NEVE, R.L., ORKIN, S.H. & BRUNS, G.A.P. (1990). Homozygous deletion in Wilms' tumours of a zinc-finger gene identified by chromosome jumping. *Nature*, **343**, 774–778.

HABER, D.A., SOHN, R.L., BUCKLER, A.J., PELLETIER, J., CALL, K.M. & HOUSMAN, D.E. (1991). Alternative splicing and genomic structure of the Wilms' tumour gene, WT1. *Proc. Natl Acad. Sci. USA*, **88**, 9618–9622.

HOGG, A., ONADIM, Z., BAIRD, P.N. & COWELL, J.K. (1992). Detection of heterozygous mutations in the RB1 gene in retinoblastoma patients using single strand conformation polymorphism (SSCP) analysis and polymerase chain reaction sequencing. *Oncogene*, **7**, 1444–1451.

HUFF, V., MIWA, H., HABER, D.A., CALL, K.M., HOUSMAN, D., STRONG, L.C. & SAUNDERS, G.F. (1991). Evidence for WT1 as a Wilms' tumour (WT) gene: intragenic germinal deletion in bilateral WT. *Am. J. Hum. Genet.*, **48**, 997–1003.

JADRESIC, L., WADEY, R.B., BUCKLE, B., BARRATT, T.M., MITCHELL, C.D. & COWELL, J.K. (1991). Molecular analysis of chromosome region 11p13 in patients with Drash syndrome. *Hum. Genet.*, **86**, 497–501.

MANNENS, M., SLATER, R.M., HEYTIG, C., BLIEK, J., DE KRAKER, J., COAD, N., DE PAGTER-HOLTHUIZEN, P. & PEARSON, P.L. (1988). Molecular nature of genetic changes resulting in loss of heterozygosity of chromosome 11 in Wilms' tumours. *Hum. Genet.*, **81**, 41–48.

NARAHARA, K., KIKKAWA, K., KIMIRA, S., KIMOTO, H., OGATA, M., KASAI, M. & MATSUOKA, K. (1984). Regional mapping of catalase and Wilms' tumour, aniridia, genitourinary abnormalities, and mental retardation triad loci of the chromosome segment 11p1305-p1306. *Hum. Genet.*, **66**, 181–185.

ONADIM, Z., HOGG, A., BAIRD, P.N. & COWELL, J.K. (1992). Oncogenic point mutations in exon 20 of the RB1 gene in families showing incomplete penetrance and mild expression of the retinoblastoma phenotype. *Proc. Natl Acad. Sci. USA*, **89**, 6177–6181.

PELLETIER, J., BRUENING, W., KASHTAN, C.E., MAUER, S.M., MANIVEL, J.C., STRIEGEL, J.E., HOUGHTIN, D.C., JUNIEN, C., HABIB, R., FOUSER, L. & 4 others (1991*a*). Germline mutations in the Wilms' tumour suppressor gene are associated with abnormal urogenital development in Denys-Drash syndrome. *Cell*, **67**, 437–447.

PELLETIER, J., BRUENING, W., LI, F.P., HABER, D.A., GLASER, T. & HOUSMAN, D.E. (1991*b*). WT1 mutations contribute to abnormal genital system development and hereditary Wilms' tumour. *Nature*, **353**, 431–434.

PRITCHARD-JONES, K. & FLEMING, S. (1991). Cell types expressing the Wilms' tumour gene (WT1) in Wilms' tumours: implications for tumour histogenesis. *Oncogene*, **6**, 2211–2220.

RICCARDI, V.M., SUJANSKY, E., SMITH, A.C. & FRANCKE, U. (1978). Chromosome imbalance in the aniridia-Wilms' tumour association: 11p interstitial deletion. *Pediatrics*, **61**, 604–610.

STANBRIDGE, E.J. (1990). Human tumor suppressor genes. *Am. Rev. Genet.*, **24**, 615–657.

TADOKORO, K., FUJII, H., OHSHIMA, A., KAKIZAWA, Y., SHIMIZU, K., SAKAI, A., SUMIYOSHI, K., INOUE, T., HAYASHI, Y. & YAMADA, M. (1992). Intragenic homozygous deletion of the WT1 gene in Wilms' tumour. *Oncogene*, **7**, 1215–1221.

TON, C.C.T., HUFF, V., CALL, K.M., COHN, S., STRONG, L.C., HOUSMAN, D.E. & SAUNDERS, G.F. (1991). Smallest region of overlap in Wilms' tumor deletions uniquely implicates an 11p13 zinc finger gene as the disease locus. *Genomics*, **10**, 293–297.

WADEY, R.B., PAL, N.P., BUCKLE, B., YEOMANS, E., PRITCHARD, J. & COWELL, J.K. (1990). Loss of heterozygosity in Wilms' tumour involves two distinct regions of chromosome 11. *Oncogene*, **5**, 901–907.

WAZIRI, M., PATIL, S.R., HANSON, J.W. & BARTLEY, J.A. (1983). Abnormalities of chromosome 11 in patients with features of Beckwith-Wiedemann syndrome. *J. Pediat.*, **102**, 873–876.

WIEDEMANN, H.R. (1983). Tumours and hemihypertrophy associated with Wiedemann-Beckwith syndrome. *Eur. J. Pediatr.*, **141**, 129.